# Schedule-dependent response of neuroblastoma cell lines to combinations of etoposide and cisplatin

**DOI:** 10.1038/sj.bjc.6600060

**Published:** 2002-02-01

**Authors:** E L Meczes, A D J Pearson, C A Austin, M J Tilby

**Affiliations:** Paediatric Oncology Laboratory, Cancer Research Unit, Catherine Cookson Building, The Medical School, The University of Newcastle upon Tyne, Newcastle upon Tyne NE2 4HH UK; Department of Child Health, Sir James Spence Inst. Royal Victoria Infirmary, Queen Victoria Road, Newcastle upon Tyne NE1 4LP, UK; Department of Biochemistry and Genetics, The Medical School, The University of Newcastle upon Tyne, Newcastle upon Tyne NE2 4HH, UK

**Keywords:** cisplatin, etoposide, synergy, neuroblastoma, chemotherapy

## Abstract

The growth inhibitory effects of cisplatin and etoposide on neuroblastoma cell lines were investigated in several scheduled combinations. Results were analyzed using median effect and combination index analyses. In all schedules in which cisplatin was administered prior to etoposide a synergistic effect was observed. Conversely, an antagonistic effect was seen in all schedules where etoposide was administered before cisplatin.

*British Journal of Cancer* (2002) **86**, 485–489. DOI: 10.1038/sj/bjc/6600060 www.bjcancer.com

© 2002 The Cancer Research Campaign

## 

The design of the widely used OPEC protocol (oncovins, platinum agents, epipodophyllotoxins and cyclophosphamide) for management of the paediatric solid tumour neuroblastoma was based on clinical and *in vitro* evaluations of sequentially scheduled cisplatin and the epipodophyllotoxin, teniposide. These studies indicated that, for optimal anti-tumour effect, cisplatin should be administered prior to the epipodophyllotoxin (Hayes *et al*, 1981; Shafford *et al*, 1984; Pritchard *et al*, 1985).

In several more recent clinical protocols for neuroblastoma, this apparently optimal order of drug administration has not been retained. The order of administration has been reversed in some regimens (Gordon *et al*, 1992; Tweddle *et al*, 2001) or the drugs are administered concurrently (reviewed in Pinkerton *et al*, 2000). If the OPEC rationale was correct, this altered scheduling pattern could result in sub-optimal response. Furthermore, in many of these schedules, cisplatin has been replaced by carboplatin. This will accentuate the change in order of administration because, despite the fact that carboplatin forms essentially the same final DNA adducts as cisplatin, the cytotoxic cross-linked structures develop more slowly than with cisplatin (Knox *et al*, 1986).

The present study was aimed at determining, using contemporary methods of analysis, whether the currently used epipodophyllotoxin, etoposide, in combination with cisplatin, caused effects on neuroblastoma cell lines that were dependent upon the relative timings of the drug exposures.

## MATERIALS AND METHODS

### Cell culture and drug solutions

The neuroblastoma cell lines SHSY5Y (Ciccarone *et al*, 1989) and NGP (Brodeur *et al*, 1977) were kindly provided by Drs P Lovat and D Tweddle (University of Newcastle upon Tyne). Cells were grown in RPMI 1640 (Dutch modification, supplemented with 10% v/v foetal bovine serum, and antibiotics (Gibco BRL)) at 37°C/5% CO_2_. Frequent tests for mycoplasma infection were always negative. Etoposide was dissolved in methanol and stored at −20°C. Cisplatin was freshly dissolved in DMSO just prior to each experiment and immediately diluted into medium. During drug exposures, the concentrations of methanol and DMSO were kept below 1% and 0.001% respectively in treated and control wells.

### Sulphorhodamine B (SRB) assay

For each experiment, cells were inoculated into three 96-well tissue culture plates and incubated for 24 h prior to starting drug exposures (time zero). Plates were exposed to graded concentrations of either: the first drug in the schedule only followed by the diluent for the second drug; the diluent for the first drug in the schedule followed by the second drug in the schedule; or both drugs in the schedule according to the schedule design. The plates were washed twice in drug free medium in between each drug/diluent exposure. After the final wash plates were returned to the incubator for 5 days. Procedures for fixing, staining and reading (OD570) were carried out as described by Skehan *et al* (1990).

### Median effect and combination index analysis

Drug interactions were analyzed using CalcuSyn (Chou and Hayball, 1996). This software calculates the median effect dose, Dm (analogous to the IC_50_), of the drug combinations using the median effect equation. Determination of synergy or antagonism was based on the multiple drug effect equation of Chou and Talalay (1977, 1983) and was quantified by the combination index (CI). CI=1 indicates an additive effect; <1, synergy, >1, antagonism. Results are shown for the mutually exclusive assumption of modes of activity of the drugs, however, applying the alternative assumption showed the same pattern of results.

## RESULTS

### Response of neuroblastoma cell lines to single agents

A prerequisite for evaluating the effect of drug combinations on cells is the determination of the effects of each agent acting alone within the schedule, retaining the exact timings of drug exposure as in the combined treatments. Effects of single agents were measured under four different schedules, which paralleled the more complex conditions of the combination experiments.

The responses of SHSY5Y cells exposed to etoposide were affected by the interval between seeding and drug exposure as is illustrated by the Dm values in Table 1

**Table 1:**
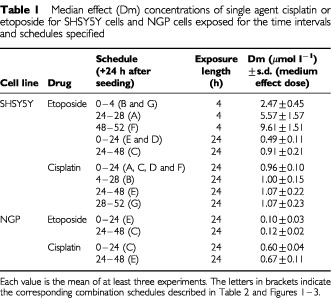
Median effect (Dm) concentrations of single agent cisplatin or etoposide for SHSY5Y cells and NGP cells exposed for the time intervals and schedules specified

. Sensitivity of the cells exposed to etoposide for 4 or 24 h periods, decreased with an increase in the interval between seeding and drug exposure.

Sensitivity of NGP cells to etoposide was not dependent on the time interval between seeding and drug exposure and was approximately 5–10-fold greater than the SHSY5Y cells (Table 1).

The responses of SHSY5Y and NGP cells to cisplatin, were independent of the time interval between seeding and drug exposure (Table 1). NGP cells were slightly more sensitive to cisplatin than SHSY5Y cells (Table 1).

### Response of SHSY5Y cells to combinations of etoposide and cisplatin

Figure 1

**Figure 1:**
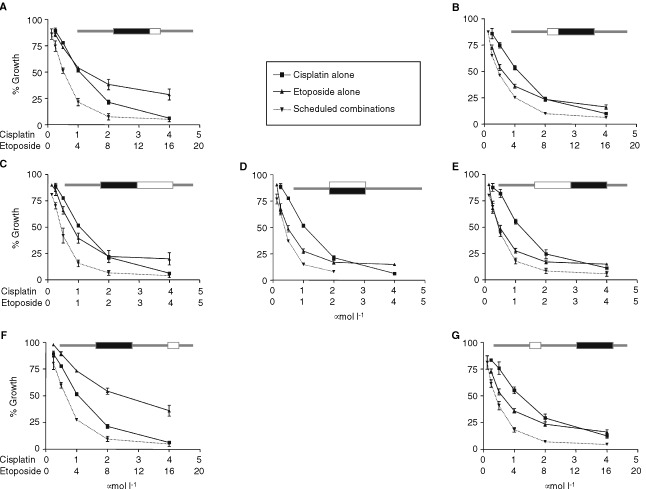
Dose response curves for SHSY5Y cells exposed to scheduled exposures of cisplatin and etoposide. For each schedule, curves are shown for both drugs as single agents administered at appropriate time points together with the drug combination. Cells were seeded in 96-well plates and allowed to adhere for 24 h (time 0). Cells were exposed to either a mixture of cisplatin and etoposide for 24 h (**D**), or, cisplatin for 24 h (**A**, **C**, **F**), washed and further exposed to 4 h of etoposide (**A**), 24 h etoposide (**C**) or drug free medium for 24 h followed by 4 h of etoposide (**F**). Panels** B**, **E** and **G** show the reverse of schedules shown in **A**, **C**, and **F** respectively. The mean of at least three experiments are shown and error bars represent standard deviations (s.d.) from the mean. For schematics at the top of each panel, black rectangles represent cisplatin exposures, white rectangles represent etoposide exposures.

shows the dose response curves for SHSY5Y cells in each of seven schedules and Figure 2

**Figure 2:**
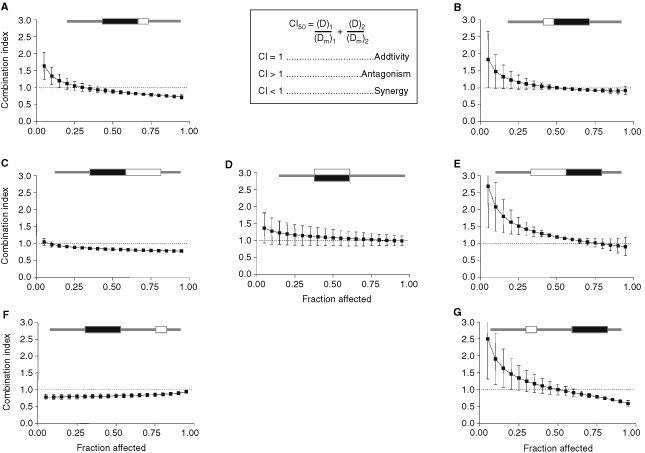
Combination index plots for SHSY5Y cells exposed to scheduled exposures of cisplatin and etoposide. Combination index plots were generated by the computer software CalcuSyn® for each of at least three experiments for each schedule. The data points in each panel represent the means of the generated plots and error bars represent the standard deviations. The equation for calculation of the combination index for two drugs (1 and 2) with mutually exclusive modes of action at the median effect dose is also shown. For schematics at the top of each panel, black rectangles represent cisplatin exposures, white rectangles represent etoposide exposures.

shows the mean combination index (CI) values plotted against fraction of cells affected. Figure 1C, D and E represent the results obtained when each drug was present for 24 h, either one immediately before the other or concurrently. There was little difference between the dose-response curves or Dm values for these schedules (Figure 1 and Table 2

**Table 2:**
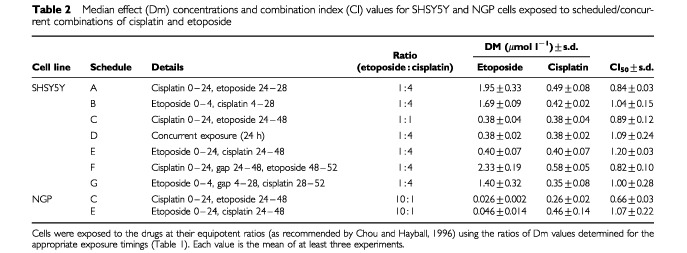
Median effect (Dm) concentrations and combination index (CI) values for SHSY5Y and NGP cells exposed to scheduled/concurrent combinations of cisplatin and etoposide

). However, the Dm values alone do not take into account the effect of the timing upon etoposide sensitivity in relation to seeding of the cells. As reported above, cells exposed to etoposide at time zero were approximately two-fold more sensitive than cells exposed 24 h later (Table 1). Combination index analysis based on single drug controls, takes this effect into account (Figure 2 and Table 2). Comparison of C, D and E in Figure 2 show a trend for the CI values to increase as the order of drug exposure changes from cisplatin first, to simultaneous exposure, to cisplatin last. For schedule C, most CI values were less than one, indicating a slight degree of synergy, while for schedule E, most values were greater than one, indicating antagonism.

In experiments aimed at more closely representing the clinical schedules of OPEC and OJEC, cisplatin exposure was for 24 h while etoposide was added for 4 h. When the drug exposures followed each other immediately, combination index values were slightly lower when cisplatin preceded etoposide (Figure 2A,B). When a 24 h period occurred between the same drug exposures, cisplatin before etoposide (schedule F), resulted in a lower degree of inhibition and higher Dm values (Table 2) compared to the opposite order (Figure 1F,G). This was the predicted result based on the difference in potency between etoposide administered at zero time and at 48 h after zero time (approximately four-fold; Table 1). However, the CI values were consistently less than 1 when exposure to cisplatin was first (Figure 2F,G), indicating that the synergistic effect of this drug sequence was enough to overcome the increased potency of etoposide when it was added at zero time.

Overall, the data in Figure 2 show that, changing schedules from cisplatin first (left-hand panels) to cisplatin last (right-hand panels) resulted in increased CI values. This indicates a consistent change from synergy to antagonism when the etoposide exposure was changed from after to before cisplatin.

### Response of NGP cells to combinations of etoposide and cisplatin

For NGP cells, the schedule in which cisplatin was administered for 24 h prior to etoposide for 24 h required significantly lower doses to achieve 50% inhibition of growth than did the reverse schedule (Table 2).

**Figure 3:**
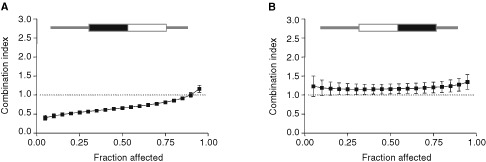
Combination index plots for NGP cells exposed to scheduled exposures of etoposide and cisplatin. The computer software CalcuSyn® simulates combination index curves to fit experimental values. For each schedule, the mean and standard errors are shown for at least three replicate experiments. The dotted line at CI=1 represents additivity. Values above this line indicate antagonism, below this line indicates synergy. (**A**) Sequential exposure to cisplatin followed by etoposide each for 24 h (Schedule C). (**B**) the reverse of A. (Schedule E).

The combination index analyses confirmed the difference between the Dm values for these schedules (Figure 3

). For schedule C, in which cisplatin was administered prior to etoposide, the majority of the CI values were less than 1, indicating synergy (Figure 3A). Conversely, for schedule E, the majority of the values were greater than 1, indicating antagonism (Figure 3B). Therefore, cisplatin prior to etoposide produced the greatest growth inhibitory effect upon NGP cells.

## DISCUSSION

Scheduling patterns in which cells were exposed to both drugs, each for 24 h were shown to result in a synergistic response in both SHSY5Y cells and NGP cells when cisplatin was administered prior to etoposide. SHSY5Y cells also exhibited this response when the exposure time to etoposide was reduced to 4 h (NGP cells were not tested with this schedule). Introducing a 24 h drug-free period between drug exposures gave a similar pattern of combination indexes to the other schedules. Overall, the results showed that changing schedules from cisplatin first to cisplatin last resulted in a change from synergy to antagonism. The exact degrees of synergy/antagonism are probably dependent upon both the model underlying the analysis method and the techniques used to measure cytotoxicity.

In the recently developed OJEC protocol for neuroblastoma therapy, carboplatin is administered for 1 h immediately following a 4 h etoposide infusion (Tweddle *et al*, 2001), allowing day-care chemotherapy. This is a reversal of the OPEC protocol in which cisplatin is infused for 24 h, followed by 24 h post-hydration and finally 4 h of etoposide. Replacement of cisplatin by carboplatin also reduces toxic side-effects. Cisplatin and carboplatin form essentially the same DNA-adduct structures (Knox *et al*, 1986), however, carboplatin monofunctional adducts are converted to bifunctional adducts much more slowly than those formed with cisplatin (Knox *et al*, 1986; Peng *et al*, 1997), further complicating analysis of synergy. This slow formation of the toxic products with carboplatin will tend to enhance the antagonistic effects of administration of the platinum agent after etoposide.

Etoposide acts via the stabilization of topoisomerase II (topo II) cleavable complexes. This effect is reversible and furthermore etoposide has a short elimination half-life (Hsiang and Liu, 1989; Slevin, 1991; Caldecott *et al*, 1993; Willmore *et al*, 1998). Together with the slow formation of carboplatin-DNA adducts, the swift reversal of etoposide stabilised topo II cleavable complexes may result in reduced anti-tumour effect in patients.

In conclusion, if the results of this study are reflected by the action of drugs in the patient, then the adoption of schedules in which platinum agent follows etoposide and in which formation of platinum-DNA cross-links is delayed (carboplatin as opposed to cisplatin) may result in sub-optimal anticancer action. An understanding of the underlying mechanisms of interaction may help assessment of the clinical relevance of these effects.
